# Supplementary figures and images for: Temperature-induced changes in egg white antimicrobial concentrations during pre-incubation do not influence bacterial trans-shell penetration but do affect hatchling phenotype in Mallards
Source: PeerJ. 2021 Nov 11;9:e12401. doi: 10.7717/peerj.12401 (PMC8590799; doi:10.7717/peerj.12401)

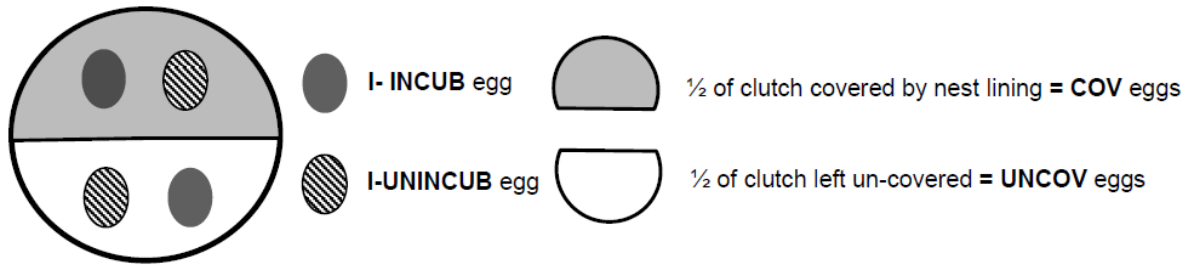

Supplement: Supplemental Information 1 — Experimental eggs (n = 160) were exposed in semi-artificial nests (n = 40) for 9 days in a natural breeding habitat. I-INCUB = partially incubated eggs, I-UNINCUB = un-incubated eggs. Note: Figure was adopted from the previous study of Javůrková et al. (2014): Ibis 156, 374–386. Copyright 2014 by Veronika Javůrková. [file peerj-09-12401-s001.pdf]

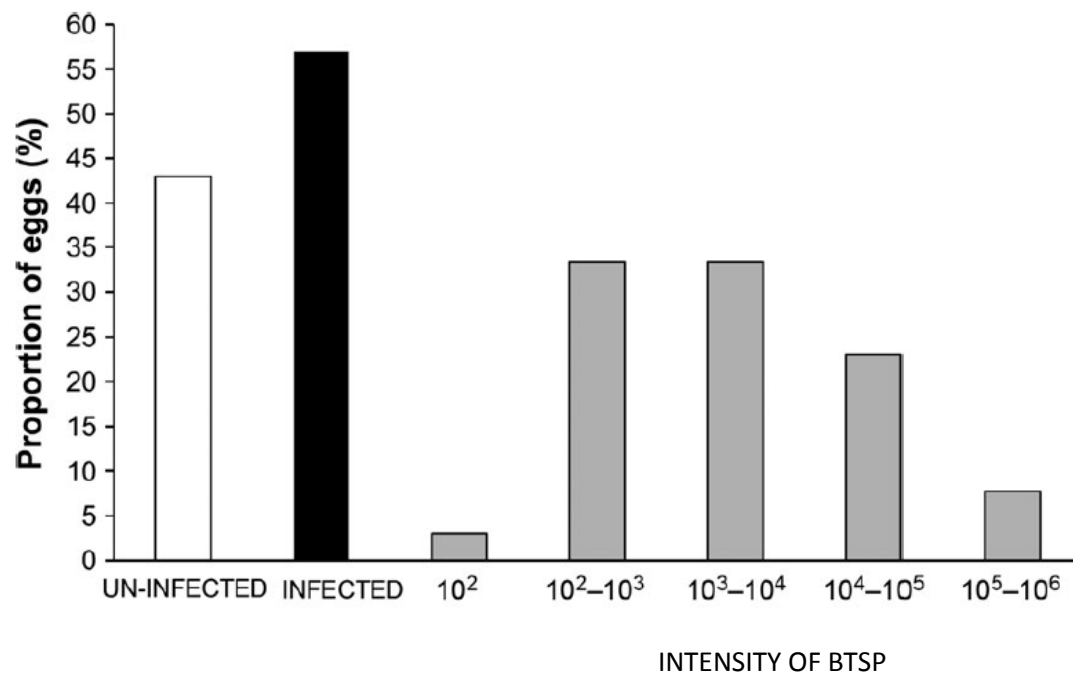

Supplement: Supplemental Information 2 — Proportion (%) of penetrated (black bar) vs. non-penetrated (white bar) experimental eggs and intensities of BTSP in penetrated eggs (grey bars) expressed as the number of bacterial cells per one mL of egg white. Note: figure was adopted from the previous study of Javůrková et al. (2014): Ibis 156, 374–386. Copyright 2014 by Veronika Javůrková. [file peerj-09-12401-s002.pdf]

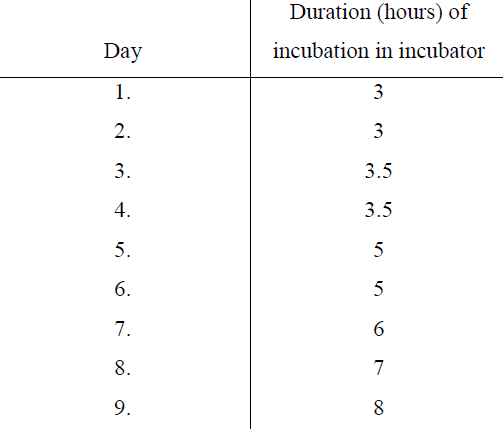

Supplement: Supplemental Information 3 — Eggs were exposed in an incubator OvaEasy 190 Advance (Brinsea Products Inc., Titusville, FL, USA) at 37.6 °C with a relative humidity of 60% for a period of 9 days. Note: table was adopted from the previous study of Javůrková et al. (2014): Ibis 156, 374–386. Copyright 2014 by Veronika Javůrková. [file peerj-09-12401-s003.docx]
